# Assessing myeloma bone disease with whole-body diffusion-weighted imaging: comparison with x-ray skeletal survey by region and relationship with laboratory estimates of disease burden

**DOI:** 10.1016/j.crad.2015.02.013

**Published:** 2015-06

**Authors:** S.L. Giles, N.M. deSouza, D.J. Collins, V.A. Morgan, S. West, F.E. Davies, G.J. Morgan, C. Messiou

**Affiliations:** a MRI Department, Royal Marsden Hospital, Sutton, Surrey, UK; b Clinical Magnetic Resonance Unit, Institute of Cancer Research, Sutton, Surrey, UK; c Haemato-oncology Department, Royal Marsden Hospital, Sutton, Surrey, UK; d Molecular Pathology, Institute of Cancer Research, Sutton, Surrey, UK

## Abstract

**Aim:**

To estimate and compare the extent of myeloma bone disease by skeletal region using whole-body diffusion-weighted imaging (WB-DWI) and skeletal survey (SS) and record interobserver agreement, and to investigate differences in imaging assessments of disease extent and apparent diffusion coefficient (ADC) between patients with pathological high versus low disease burden.

**Materials and methods:**

Twenty patients with relapsed myeloma underwent WB-DWI and SS. Lesions were scored by number and size for each skeletal region by two independent observers using WB-DWI and SS. Observer scores, ADC, and ADC-defined volume of tumour-infiltrated marrow were compared between patients with high and low disease burden (assessed by serum paraproteins and marrow biopsy).

**Results:**

Observer scores were higher on WB-DWI than SS in every region (*p*<0.05) except the skull, with greater interobserver reliability in rating the whole skeleton (WB-DWI: ICC = 0.74, 95% CI: 0.443–0.886; SS: ICC = 0.44, 95% CI: 0.002–0.730) and individual body regions. WB-DWI scores were not significantly higher in patients with high versus low disease burden (observer 1: mean ± SD: 48.8 ± 7, 38.6 ± 14.5, observer 2: mean ± SD: 37.3 ± 13.5, 30.4 ± 15.5; *p* = 0.06, *p* = 0.35).

**Conclusion:**

WB-DWI demonstrated more lesions than SS in all regions except the skull with greater interobserver agreement. Sensitivity is not a limiting factor when considering WB-DWI in the management pathway of patients with myeloma.

## Highlights

• WB-DWI demonstrated significantly more lesions than SS in all body regions except the skull.
• Inter-observer agreement was better when assessing WB-DWI compared to SS.
• Sensitivity is not a limitation of WB-DWI in myeloma.

## Introduction

In myeloma, a plasma cell disease with diffuse or focal marrow infiltration, skeletal involvement is often heterogeneous and variable.^1,2^ Disease burden is assessed by serum markers and bone trephine,^3^ but these tests are not always reliable: bone trephine is prone to sampling error and neither test provides information on the extent and distribution of disease. In asymptomatic patients the skeleton is imaged with a skeletal survey (SS) as bone disease is a criterion of symptomatic myeloma that requires treatment.^4^ However, despite the low sensitivity of SS, which is limited to imaging the secondary effects of disease on cortical bone,^5–9^ its low cost and widespread availability mean that it remains the recommendation of the International Myeloma Working Group (IMWG) as a first-line screen for bone involvement.^1,10,11^ For patients with bone pain, some centres are substituting skeletal survey with whole-body low-dose CT, but due to limited contrast within the marrow itself, this too lacks sensitivity.^12–14^ Current guidelines suggest MRI of the spine in asymptomatic patients where SS or CT are negative and a positive MRI in an asymptomatic patient is increasingly recognized as an indication to treat.^15–17^

Whole-body diffusion-weighted MRI (WB-DWI) is now recognized as a promising clinical tool because it can provide information on differences between normal and diseased bone marrow microarchitecture^18^ for the entire skeleton in a 25 min time frame. Significant differences in the measured marrow apparent diffusion coefficient (ADC) between normal subjects and myeloma patients^18,19^ and between patients with active myeloma and those in remission^20^ have been demonstrated. Because it provides a means of quantification, WB-DWI in myeloma also has been explored as a measure of response.^21^ The only other quantitative imaging technique investigated in myeloma is^18^FDG-PET/CT, sensitivity and specificity of which is inferior to even conventional MRI^1,22,23^ with skull and rib lesions poorly demonstrated in both cases.^24^ In addition, ^18^FDG-PET/CT carries a significant cost implication as well as a radiation dose. This makes WB-DWI potentially more attractive for staging and response assessment because not only can disease be assessed by skeletal region, but it can also be related to pathological disease burden. Therefore, the aims of the present study were to estimate and compare the extent of myeloma bone disease by skeletal region using WB-DWI and SS, assess interobserver agreement of both techniques, and investigate differences in imaging assessments of disease extent and ADC between patients with a pathological high versus low disease burden.

## Materials and methods

This was a prospective, HIPAA (Health Insurance Portability and Accountability Act) compliant, single-institution study with approval from the local Research Ethics Committee. Written informed consent was obtained from each patient.

### Patients and volunteers

Twenty patients with relapsed active myeloma (aged 45–73 years, eight male, 12 female) underwent WB-DWI and SS prior to starting treatment. Patients with relapsed myeloma were recruited in order to ensure the presence of active disease at multiple skeletal sites. Patients with suspected spinal cord compression or contra-indications to MRI were excluded. Serum paraproteins, light chains, and bone marrow histology were recorded to assess patients' pathological disease burden, defined according to current guidelines.^11^ Patients were classified as having a high burden of disease if there was ≥50% infiltration of plasma cells on bone marrow trephine; otherwise they were classified as low burden of disease. Clinical characteristics of the patients, including the presence or absence of CRAB features (hypercalcaemia: corrected serum calcium >0.25 mmol/l above upper limit of normal or >2.75 mmol/l; renal insufficiency: serum creatinine >173 μmol/l; anaemia: haemoglobin 2 g/dl below lower limit of normal or <10 g/dl; bone lesions: present),^25^ are summarized in Table 1. Follow-up laboratory assessments after three cycles of treatment showed a response to treatment in 14 of 20 patients and progressive disease in three of 20 (classified according to the IMWG uniform response criteria),^3^ confirming active disease at the outset. In the remaining three patients with stable disease, changes in serum paraproteins did not reach criteria for partial response or progressive disease.

### Image acquisition

Using an Avanto 1.5 T system (Siemens, Erlangen, Germany) a WB study was achieved by the serial acquisition of contiguous body regions. All participants were scanned supine with arms by their sides. Coil elements were positioned from skull vertex to knees. Axial T1-weighted (W) spin-echo [5 mm section thickness, no gap, 430 mm field of view (FOV), anteroposterior (AP) phase direction, 386 ms repetition time (TR)/ 4.8 ms echo time (TE), 70° flip angle, 256 × 154 matrix] and coronal VIBE Dixon 3D gradient-echo breath-hold sequences (52 sections per slab, 470 mm FOV, 7 ms TR/2.38,4.76 ms TE, 3° flip angle, 192 × 192 matrix) were acquired, followed by axial DW sequences [single-shot double spin-echo echo-planar technique with short tau inversion recovery (STIR) fat suppression in free breathing]. b-Values of 50 and 900 s/mm^2^ were applied in three orthogonal directions and combined to provide isotropic trace images. DW sequences were acquired in blocks of 50 sections (5 mm section thickness, no gap, 430 mm FOV, AP phase direction, parallel imaging (GRAPPA) factor 2, 14,800 ms TR, 66 ms TE, 180 ms inversion time (TI), 2.9 × 2.9 × 5 mm voxel size, four signal averages acquired, 150 × 150 matrix, 1960 Hz per pixel bandwidth). The optimized scanner carrier frequency offset used for the top station was applied for all other stations.^26^ The same shim gradient currents were applied for each station.^27^ Total acquisition time was 50–60 min.

SS radiographs were acquired using a Carestream Digital Radiography system (Rochester, NY, USA) and consisted of the following series of projections: lateral skull, posteroanterior (PA) chest, AP pelvis, AP and lateral cervical (C), thoracic (T) and lumbar (L) spines, AP humerii, AP femora.

### Image analyses

Morphological images were checked for the presence of fractures and other benign lesions, such as significant vertebral haemangiomas, which have the potential to affect the diffusion-weighted signal. No such lesions were noted in this small cohort.

#### Observer scores

For each body region (skull, C spine, T spine, L spine, pelvis, ribs/other, long bones) two radiologists (C.M. and N.D.S.) with 7 years of experience of DWI in bone (blinded to clinical information) made a categorization of disease burden with a previously used scoring system.^21^ This was based on number of lesions (diffuse, >20, 10–20, <10, 0) and largest lesion dimension (diffuse, >10, 5–10, <5, 0 mm) on WB-DW images, assigning a score from 4 to 0 for each characteristic (lesion number and size).^21^ Images provided were b = 50 s/mm^2^ and b = 900 s/mm^2^ source images, ADC maps and composed WB-DW inverted greyscale maximum intensity projection (MIP) b = 900 s/mm^2^ images. All images were reviewed in conjunction as T2 shine through from old inactive sites at b = 900 s/mm^2^ can mimic cellular active disease. On a separate occasion, at least 2 weeks apart, the same two observers used the same system to categorize lesions observed on SS. The same image scoring system was then applied to each categorization, so that the possible score for each body region on WB-DWI and SS ranged from 8-0, (56-0 for the whole skeleton).

#### ADC derivation

Quantitative ADC analysis was undertaken using OncoTreat software (Siemens, Erlangen, Germany). Multiple volumetric regions of interest were outlined against three-dimensional multiplanar reformatted (MPR) images of the b = 900 s/mm^2^ data using a semi-automated technique, whereby one set of “seeds” was manually placed inside every region to be included in the analyses, with a second set being defined to exclude surrounding areas. The software then generated outlines of volumes to be included in the segmentation, based on signal intensity values. Segmentations included all areas of visible marrow within vertebral bodies, pelvis, femora, proximal humeri, and sternum, and were undertaken by one observer (S.G.). ADC values for every voxel within the segmented volume were recorded and displayed as histograms. The volume of tumour infiltrated marrow was defined by recording every voxel with an ADC ≥774 but ≤1433 mm^2^/s^18,28^ and compared against laboratory measures of disease burden.

### Statistical analyses

#### Comparison of WB-DWI and SS

Paired *t*-tests were used to evaluate whether each observer assessed that WB-DWI scores were significantly different from SS scores by body region and per patient. A value of *p*<0.05 was chosen as the criterion for statistical significance in all tests. Scores assigned to WB-DWI and SS were also compared between observers by calculating the intraclass correlation coefficient (ICC) for interobserver reliability for whole skeleton and individual body areas using MedCalc software (Version 14.12.0, Ostend, Belgium).

#### Comparison of imaging and pathological estimates of disease burden

Normality plots and the Kolmogorov–Smirnov and Shapiro–Wilk tests were used to confirm normality using SPSS for Windows software (Version 20, SPSS, IBM, New York, NY, USA). Independent samples *t*-tests were used to determine whether the distribution of observer scores, ADC metrics, and tumour volume were different in patients with a high or low disease burden. In addition, Pearson's correlation coefficients were calculated to evaluate the strength of any relationship between observer scores, measured ADC metrics, and laboratory measures of disease burden in the patients.

## Results

### Comparison of WB-DWI and SS

**Figure 1:**
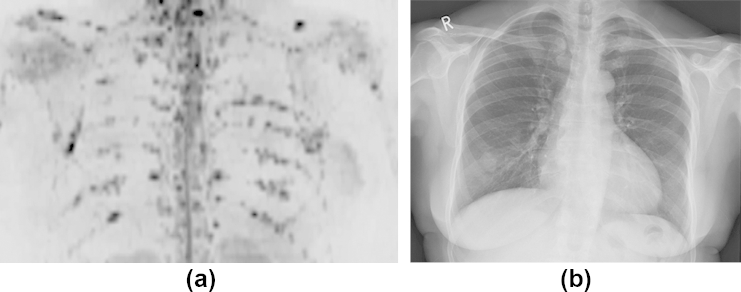
Images of the chest in a 65-year-old female patient with non-secretory myeloma shown by (a) inverted greyscale b = 900 s/mm^2^ DW images and (b) chest radiograph. Numerous discrete focal lesions are visualized in the ribs on the DWI, which are not easily seen on the chest radiograph.

Observer scores for WB-DWI and SS for whole skeleton and by body region are given in Table 2. For observer 1, WB-DWI scores were higher than SS scores in 16 of 20 patients, equivalent for two patients and lower in two patients. For observer 2, WB-DWI scores were higher than SS scores in 19 of 20 patients and lower in one patient. When assessed by region, both observers scored WB-DWI significantly more highly than SS in every region (*p*<0.05) except the skull, where observer 1 scored DWI more highly (*p* = 0.03) but observer 2 did not (*p* = 0.8). There were significantly more patients with a positive score (>0) in each region outside the skull on WB-DWI than on SS for both observers (Table 3, Fig 1). There was greater interobserver reliability in rating WB-DWI than SS scores when assessed for the whole skeleton (WB-DWI: ICC = 0.74, 95% CI: 0.443–0.886; SS: ICC = 0.44, 95% CI: 0.002–0.730) or by region (Table 4).

### Comparison of imaging and pathological estimates of disease burden

Of the 20 patients, six were classified as having a high burden of disease and 12 as low burden (in two patients bone marrow samples were unquantifiable). None of the patients had a raised serum calcium level; two exhibited renal insufficiency (one in the low disease burden group and one where there was no quantified bone marrow sample), and five were anaemic (one high burden of disease, two low burden of disease, two unquantified samples).

#### Observer scores

**Figure 2:**
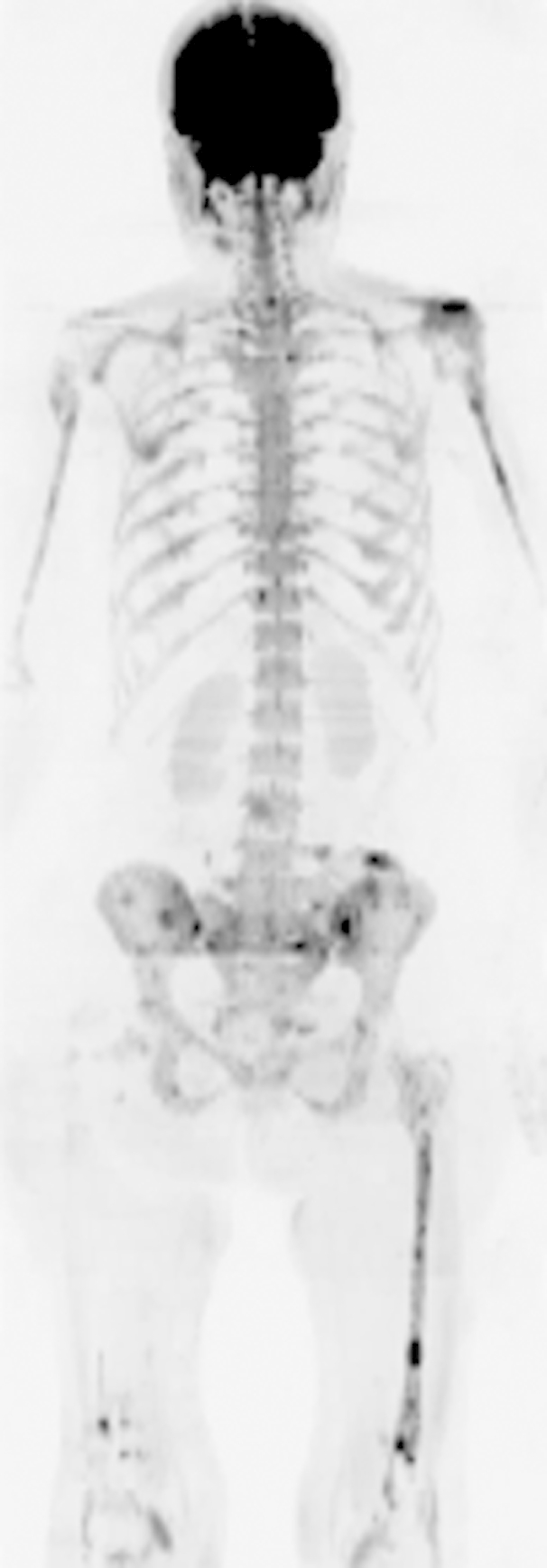
Inverted greyscale b = 900 s/mm^2^ WB-DW images in a 65-year-old female patient with myeloma and a high disease burden of 70% clonal cells on bone marrow biopsy (note right femoral nail).

**Figure 3:**
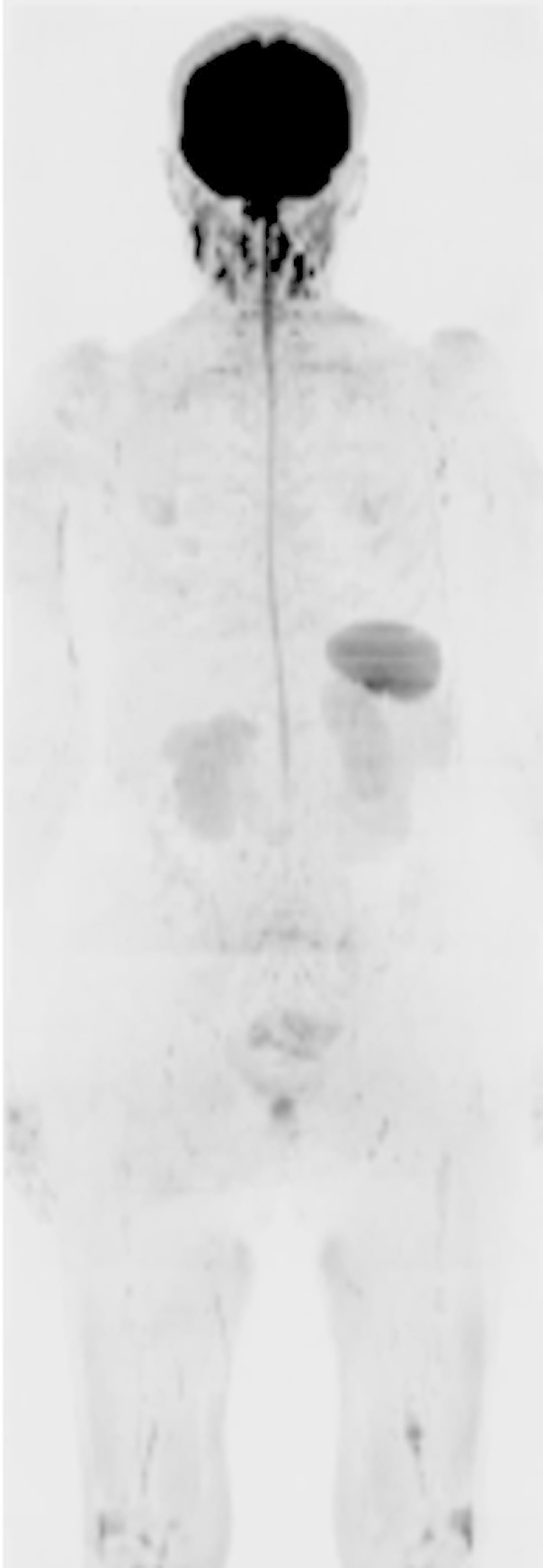
Inverted greyscale b = 900 s/mm^2^ WB-DW images in a 60-year-old female myeloma patient with myeloma and a low disease burden of 5% clonal cells. Note the lower signal-intensity in the skeleton compared to Fig. 2.

Mean WB-DWI scores per patient were higher in those with a high burden of disease than in those with a low burden of disease (observer 1: mean ± SD: 48.8 ± 7, 38.6 ± 14.5; observer 2: mean ± SD: 37.3 ± 13.5, 30.4 ± 15.5; Figs 2–3), but these differences did not achieve statistical significance (*p* = 0.06, *p* = 0.35 respectively). SS scores were also higher in those with a high burden of disease compared to those with a lower burden for both observers (observer 1: mean ± SD 30.2 ± 14.5, 15.1 ± 12.8; observer 2: mean ± SD 14 ± 9.3, 7.1 ± 6.3), but also did not achieve statistical significance (*p* = 0.06, *p* = 0.142 respectively). There were no significant correlations between WB-DWI observer scores and the proportion of plasma cells on bone marrow trephine (observer 1: *r* = 0.378, *p* = 0.122; observer 2: *r* = 0.227, *p* = 0.366) or between WB-DWI scores and serum paraprotein concentration (observer 1: *r* = 0.443, *p* = 0.086; observer 2: *r* = 0.474, *p* = 0.064).

#### ADC analysis

Two patients were not evaluable by ADC: in one the WB-DWI was severely degraded by artefacts, and in the other lack of visible bone marrow precluded successful segmentation. Mean marrow ADC for the whole segmented volume in each patient ranged from 659–971 × 10^−6^ mm^2^/s (mean ± SD 802 ± 89 × 10^−6^ mm^2^/s). Volume of marrow segmented in the patients ranged from 135–1338 cm^3^ (mean ± SD: 650 ± 342 cm^3^). The volume of tumour infiltrated marrow ranged from 35–555 cm^3^ (mean ± SD: 241 ± 155 cm^3^). There were no significant differences in mean ADC or volume of tumour infiltrated marrow between those classified with a low or high disease burden (*p* = 0.97, *p* = 0.80 respectively). In addition, there was no significant correlation between mean ADC or the segmented tumour volume and the proportion of plasma cells on bone marrow trephine (*r* = –0.1, *p* = 0.72, *r* = –0.04, *p* = 0.88) or with serum paraprotein concentration (*r* = –0.2, *p* = 0.52, *r* = 0.16, *p* = 0.59).

## Discussion

The present study demonstrates increased scores derived from WB-DWI compared to SS in all skeletal regions except the skull and highlights the increased sensitivity of WB-DWI. Although histological verification that the additionally detected lesions represent active sites is not feasible, other studies with histological validation have shown that lesions detected by modern imaging techniques do represent active myeloma bone lesions.^24^ Outside the skull, sites detected on SS are not missed on WB-DWI. This builds on other data comparing T1W and T2W MRI with SS where MRI showed increased sensitivity.^29–31^ The additional advantage of WB-DWI is its demonstration of more lesions in the ribs, long bones, and shoulders compared to SS.^24^ WB-DWI provides additional advantages in being able to detect extramedullary soft-tissue disease (although none was found in the present series) and to age vertebral compression fractures as both the b = 50s/mm^2^ images and ADC maps can be used to detect oedema.

In common with other studies,^24,31,32^ DWI appeared less sensitive than SS to detect disease in the skull, possibly due to obscuration from the diffusion restriction from the brain. It is also feasible that cases where SS was positive and DWI negative represented fixed cortical defects from old treated disease (as the present cohort all had relapsed disease) or prominent venous lakes. The improved observer reliability in rating WB-DWI than SS scores did not apply in the skull, partly because there were five patients in whom observer 1 rated diffuse disease in the skull but observer 2 rated no disease. The only other region in which agreement in scores was lower on DWI than SS was in the ribs, although there was agreement in the presence or absence of disease in 17 of 20 patients on DWI and in only eight of 20 on SS. Greater experience of WB-DWI is likely to improve results further, unlike SS where observer agreement remains a limitation of the technique despite years of experienced interpretation.^7^ Furthermore, the conventional MRI that is also acquired as part of the total WB examination provides additional information to complement the DWI, improve diagnostic confidence, and reduce false-positive findings from benign lesions, e.g., haemangiomas.

In the event of equivocal findings on WB MRI, the lack of any ionizing radiation makes it reasonable to acquire an interval scan for clarification. In contrast, a typical SS may confer a dose of approximately 1.8 mSv each time for each patient^33^; therefore, representing a significant potential total dose if used on a serial basis. Unfortunately, the cost of a WB MRI examination remains significantly higher than SS. However, as WB-DWI techniques develop, it is anticipated that sequence acquisition times will reduce and the degree of automated post-processing of images will increase, thereby allowing for the possibility of reduced costs for WB-DWI.

The differences in WB-DWI scores between those with a high burden of disease, compared to those with a low burden of disease at histopathology were limited by histopathological validation from a single biopsy site. Previous studies have shown that ADC can be used to differentiate patients with active myeloma from those in remission.^20^ However, the failure to demonstrate a significant relationship between ADC and laboratory measures of disease burden may well reflect the overlap between the ADC values of diffuse disease and normal marrow, compared to the greater distinction between focal disease and normal marrow.^18^ In addition, the laboratory measures of disease burden may not be representative of the extent or histological severity of disease,^34^ because of the sampling error and serum markers are not always reliable. Other contributing factors to the lack of difference in ADC between high and low pathology disease burden groups is the small size of the patient cohort as well as the uncharacterized effects of previous treatments. Further study of a larger cohort of patients, or of those newly diagnosed and not exposed to previous treatments, may help to clarify any differences between ADC in different pathological classes.

A limitation of the present evaluation is the lack of a universally accepted scoring system. Various methods have been described: Bannas et al.^35^ defined imaging response categories based on changes in size or number of lesions, whereas Hillengass et al.^36^ assessed both the number of focal lesions and the degree of diffuse infiltration. In the present study a scoring system that incorporated both of these elements was utilized.^21^ However, the authors' experience over the last 4 years has been that qualitative analysis alone is usually sufficient to assess the presence and extent of myeloma bone disease using WB-DWI, with quantitative analyses offering some advantages for assessing response to treatment.^21^

A limitation of the present ADC analyses is the resolution of the segmentation method chosen, where the most significant factor was lack of contrast in the b = 900 s/mm^2^ images in patients with little active disease. Not only was seed placement more difficult, but the subsequently generated outlines of the segmentations conformed less tightly to the borders of the bony anatomy than in patients with higher contrast images. This meant that some pixels included within the segmentations probably lay outside the target anatomy when image contrast was poor. In addition, the ribs were not included in the segmentations due to the complexity of defining seeds to separate each rib accurately and the processing power of the software. Therefore, in some patients with the highest burden of disease, or in those with disease in the ribs, the segmentation may not have been truly representative. One method for addressing this would be to define smaller volumes as separate segmentations, e.g., by outlining each vertebral body, rib, or other structure separately, but this could be very time consuming, requiring improvements in speed of software or the degree of automation to be a viable clinical option for analysing data. It is also possible that the ADC range defined by Padhani et al.^28^ to represent myeloma infiltrated marrow (774–1433 × 10^−6^ mm^2^/s) was inappropriate for this volumetric segmentation method of ADC analysis. However, the present DWI acquisition strategy was very similar to that used by Padhani et al.^28^ and the mean ± SD marrow ADC for the myeloma patients in the present study (802.2 ± 89.1 × 10^−6^ mm^2^/s) was comparable (875 ± 187 × 10^−6^ mm^2^/s). Furthermore, inspection of the present data revealed that applying other thresholds to define the tumour infiltrated marrow did not elicit any further significant findings.

In conclusion, the present study shows that WB-DWI demonstrates a greater extent of disease in myeloma patients than SS in every body region except the skull. More lesions were also seen in the ribs on DWI than SS, which has previously been a limitation of conventional MRI techniques. The improved interobserver reliability afforded by WB-DWI in the present study is also an important finding, although there is still room for improvement as experience with the technique grows. The role of WB-DWI in the routine investigation of patients with myeloma has not been established but the significant promise for imaging and quantifying response to treatment^21^ is likely to lead to further trials. This study indicates that sensitivity will not be a limiting factor when considering WB-DWI in the management pathway of patients with myeloma.

## Figures and Tables

**Table 1:** Clinical characteristics of the patients, indicating the degree of marrow infiltration on bone marrow trephine, serum paraprotein and light chain concentrations, and the range of CRAB features present in this cohort.

Patient no.	Age (years)	Sex	Myeloma type	Affected cells on BMT (%)	Serum M-protein (g/l)	Serum free κ (mg/l)	Serum free λ (mg/l)	Corrected serum Calcium (mmol/l)	Serum creatinine (μmol/l)	Haemoglobin (g/dl)	Bone lesions
1	61	M	Non Secretory	60	0	<6	5	2.19	70	12.2	N
2	65	F	κ light chain	80	0	2100	<5	2.41	77	9.3	Y
3	70	F	IgG κ	25	13	1175	<5	2.20	55	12.9	Y
4	72	F	IgG κ	20-25	20	ND	ND	2.40	78	14.0	N
5	61	M	IgG κ	ND	13	56	<5	2.24	104	8.4	Y
6	67	M	IgA λ	25-35	37	10	5000	2.50	262	10.1	Y
7	60	F	IgG λ	2-5	6	<4	37	2.31	51	12.8	N
8	67	M	IgG κ	80	8	15	9	2.30	136	13.8	Y
9	65	M	IgG λ	10-20	0	7	1275	2.16	87	12.4	Y
10	55	F	λ light chain	20-30	0	7	650	2.44	59	11.2	Y
11	62	F	IgG κ	25	13	ND	ND	2.30	65	11.6	N
12	65	F	IgG κ	60-70	49	ND	ND	2.28	83	10.4	Y
13	68	F	IgG κ	10-20	28	ND	ND	2.29	63	10.6	Y
14	68	M	IgG κ	60	39	625	6	2.61	82	11.6	Y
15	73	M	IgG κ	ND	26	ND	ND	1.96	272	9.2	Y
16	47	M	IgG κ	30-40	10	ND	ND	2.18	50	13.3	Y
17	68	F	IgG λ	40-50	23	ND	ND	2.23	40	13.1	Y
18	45	F	IgG λ	70-80	28	<4	130	2.17	58	10.4	Y
19	47	F	IgG κ	30-40	35	ND	ND	2.42	60	11.4	N
20	69	F	IgG κ	10-15	23	ND	ND	2.32	116	9.9	Y

CRAB: Hypercalcaemia defined as corrected serum calcium >0.25 mmol/l above upper limit of normal or >2.75 mmol/l; renal insufficiency if serum creatinine >173 μmol/l; anaemia if haemoglobin 2 g/dl below lower limit of normal or <10 g/dl; bone lesions defined by ≥ 1 lytic lesion seen on SS departmental report. BMT, bone marrow trephine; ND, no data.

**Table 2:** Observer scores for whole-body diffusion-weighted imaging (WB-DWI) were significantly higher than for x-ray skeletal survey (SS) for whole skeleton and in every body region outside of the skull.

Region	Observer 1 scores (mean ± SD)		Observer 2 scores (mean ± SD)		WB-DWI & SS Paired *t*-test (*p*)	
	WB-DWI	SS	WB-DWI	SS	Obs 1	Obs 2
Skull	5.3 ± 3.5	3.1 ± 2.3	2.3 ± 2.9	2.5 ± 1.6	0.03	0.83
C spine	5.8 ± 2.7	3.3 ± 3.5	4.6 ± 3.3	0.7 ± 1.2	0.04	<0.0001
D spine	6.9 ± 1.9	5.2 ± 3.5	6.5 ± 2.1	0.8 ± 1.6	0.04	<0.0001
L spine	6.5 ± 2.1	3.8 ± 3.6	4.8 ± 3.2	1.0 ± 1.4	0.003	0.0002
Pelvis	5.8 ± 2.6	2.4 ± 2.9	5.1 ± 2.7	1.4 ± 1.9	0.0001	<0.0001
Ribs/other	7.3 ± 1.3	2.8 ± 2.7	6.1 ± 3.0	1.3 ± 1.7	<0.0001	<0.0001
Long bones	5.4 ± 2.8	2.1 ± 2.0	4.9 ± 2.9	1.7 ± 1.6	0.0002	0.0001
Whole skeleton	42.4 ± 12.8	22.2 ± 15.5	33.9 ± 14.4	9.1 ± 7.7	<0.0001	<0.0001

**Table 3:** Patients scored positively on whole-body diffusion-weighted imaging (WB-DWI) and x-ray skeletal survey (SS) by each observer, by body region.

Region	Observer 1 % pts scoring positively (no. pts)			Observer 2 % pts scoring positively (no. pts)		
	WB-DWI	SS	% Difference WB-SS	WB-DWI	SS	% Difference WB-SS
Skull	75 (15)	75 (15)	0	55 (11)	80 (16)	-25
C spine	95 (19)	60 (12)	35	80 (16)	35 (7)	45
D spine	100 (20)	75 (15)	25	100 (20)	20 (4)	80
L spine	95 (19)	60 (12)	35	95 (19)	35 (7)	60
Pelvis	90 (18)	50 (10)	40	90 (18)	40 (8)	50
Ribs/other	100 (20)	65 (13)	35	85 (17)	40 (8)	45
Long bones	85 (17)	65 (13)	20	85 (17)	60 (12)	25
Whole skeleton	100 (20)	85 (17)	15	100 (20)	85 (17)	15

Pts, points.

**Table 4:** Inter-observer reliability in rating whole-body diffusion-weighted imaging (WB-DWI) and x-ray skeletal survey (SS) scores by body area and for whole skeleton.

Region	Inter-observer reliability(ICC [95% Confidence Interval])	
	WB-DWI	SS
Skull	0.22 [-0.245 to 0.607]	0.75 [0.463 to 0.897]
C Spine	0.77 [0.490 to 0.903]	0.174 [-0.292 to 0.574]
D Spine	0.34 [-0.110 to 0.673]	0.18 [-0.273 to 0.570]
L Spine	0.49 [0.069 to 0.760]	0.26 [-0.190 to 0.625]
Pelvis	0.48 [0.058 to 0.755]	0.47 [0.044 to 0.749]
Ribs/Other	0.40 [-0.037 to 0.711]	0.63 [0.270 to 0.835]
Long Bones	0.78 [0.524 to 0.907]	0.55 [0.160 to 0.796]
Whole Skeleton	0.74 [0.443 to 0.886]	0.44 [0.003 to 0.730]
